# What factors are associated with functional impairment among the oldest old?

**DOI:** 10.3389/fmed.2022.1092775

**Published:** 2022-12-22

**Authors:** André Hajek, Hans-Helmut König

**Affiliations:** Department of Health Economics and Health Services Research, University Medical Center Hamburg-Eppendorf, Hamburg Center for Health Economics, Hamburg, Germany

**Keywords:** aged 80 years and over, depression, functional impairment, cognitive impairment, income, poverty

## Abstract

**Purpose:**

Most of the existing studies did not explicitly focus on the oldest old who are at high risk of functional impairment. Moreover, some potential risk factors (such as financial poverty) of functional impairment have been neglected so far. Thus, our aim was to clarify the determinants (with a particular emphasis on financial poverty) of functional impairment exclusively among the oldest old.

**Methods:**

Data were taken from the “Survey on quality of life and subjective well-being of the very old in North Rhine-Westphalia (NRW80+)” – a representative sample of individuals ≥80 years (community-dwelling and in institutionalized settings) in North Rhine-Westphalia (*n* = 1,863, average age was 86.5 years, ranging from 80 to 102 years). Common tools were used to quantify functional impairment. In regression analysis, these determinants were included: sex, age, marital status, educational level, income poverty, asset poverty, depressive symptoms, cognitive impairment, and the number of chronic conditions.

**Results:**

Multiple linear regressions showed that higher functional impairment was associated with being female (ADL, β = 0.06, *p* < 0.01; IADL, β = 0.09, *p* < 0.01), higher age (ADL, β = 0.02, *p* < 0.001; IADL, β = 0.04, *p* < 0.001), low education (compared to high education: IADL, β = −0.10, *p* < 0.05), the presence of income poverty (ADL, β = 0.09, *p* < 0.05; IADL, β = 0.16, *p* < 0.01), more depressive symptoms (ADL, β = 0.12, *p* < 0.001; IADL, β = 0.14, *p* < 0.001), higher cognitive impairment (ADL, β = −0.03, *p* < 0.001; IADL, β = −0.06, *p* < 0.001), and a higher number of chronic conditions (ADL, β = 0.03, *p* < 0.001; IADL, β = 0.05, *p* < 0.001).

**Conclusion:**

Several determinants of functional impairment among the oldest old have been identified (i.e., being female, higher age, low education, presence of income poverty, more depressive symptoms, higher cognitive impairment, and more chronic conditions). Such knowledge (e.g., regarding the association between income poverty and functional impairment) may assist in characterizing individuals aged 80 years and over at high risk for functional impairment. Ultimately, such knowledge may help to design specific interventions for high risk groups. Moreover, such knowledge may enrich the research areas addressing inequalities.

## 1 Introduction

Limitations in basic (ADL; e.g., eating, walking, or bathing) or quite complex instrumental activities of daily living (IADL; e.g., doing housework or arranging financial matters) are also known as functional impairment. Functional impairment is strongly related to adverse factors such as admission to nursing home (1) or mortality (2, 3). Higher age is associated with a higher risk of functional impairment (4). Thus, in light of the demographic aging, it can be assumed that the number of individuals with functional impairment will increase in the mid- and long-term.

Several “classical” risk factors of functional impairment have been identified such as sociodemographic [e.g., being female or higher age (5)] or health-related factors [e.g., cognitive impairment (5), depressive symptoms (6), or multiple chronic conditions (7)]. However, most of the existing studies did not explicitly focus on the oldest old (i.e., 80 years and over) who are at high risk of functional impairment (8). Moreover, some potential risk factors of functional impairment have been neglected so far. For example, there is a complete lack of quantitative studies examining the association between financial poverty (in terms of income and asset poverty) and functional impairment. Thus, our aim was to examine the determinants (with a particular emphasis on financial poverty) of functional impairment exclusively among the oldest old. First, such knowledge can contribute to a better understanding of the determinants of functional impairment among the oldest old – which in turn can help to delay adverse consequences listed above. Second, such knowledge may enrich the research areas that address inequalities (e.g., when financial poverty is associated with a higher risk of functional impairment).

## 2 Methods

### 2.1 Sample

The data for our study are from the “Survey on quality of life and subjective well-being of the very old in North Rhine-Westphalia (NRW80+).” The representative survey was conducted from August 2017 to February 2018 in the most populous German federal state (Nord Rhine-Westphalia; with men and individuals aged 85+ being oversampled). This study included multiple subjects (health-related factors, media consumption, socioeconomic factors, etc.). The main inclusion criteria were: to be of primary residence in that state (i.e., North-Rhine Westphalia; including individuals in private households and individuals in institutional care) and to be 80 years of age or older.

The gross sample equaled 8,040 individuals. The net sample consisted of 1,863 individuals (thereof: 1,687 self-reported interviews and 176 proxy interviews). Thus, the response rate was about 23%, with inability to participate due to health reasons (1,186 individuals) and unwillingness to participate (2,993 individuals) being the key reasons for non-participation. However, the response rates need to be put into perspective with respect to the age group of participants, the setting including individuals in nursing homes, and an average interview time of approximately 90 min. Furthermore, gender, age group, living situation, and other factors were not associated with the probability of non-response (9). For more details, see Wagner et al. (9). However, it may be the case that some groups are underrepresented (e.g., individuals with a migration background).

The NRW80+ study was approved by the Ethics Committee of the Medical Faculty of the University of Cologne (No. 17-169). Informed consent was obtained from all participants or their legal representatives.

### 2.2 Outcome: Functional impairment

In our study, we first used a ADL tool [modified version of Katz et al. (10)] to quantify functional impairment. This tool had seven items [from 0 (only possible with help) to 2 (no help needed)]. It covered these areas: eating, dressing/undressing, personal hygiene, walking, getting up from bed and lying down, bathing/showering, reaching the toilet in time (reversely coded). All items were averaged and subsequently we reversed the coding. Consequently, the final score ranges from 0 to 2, with *higher* values reflecting *higher* functional impairment.

Secondly, we used a modified Lawton and Brody IADL tool to quantify functional impairment (11). This tool also had seven items [always ranging from 0 (only possible with help) to 2 (no help needed)] covering the areas: use phone, organize routes outside walking range (bus and cab), buy your own food and clothes, prepare own meals, doing housework, taking medications, and arranging financial matters. Again, all items were averaged, thereafter we reversed the coding: 0 to 2, thus *higher* values reflecting *higher* functional impairment.

### 2.3 Independent variables

The independent variables (socioeconomic and health-related factors) which were included in regression analysis were selected based on prior research (5, 7) and based on theoretical reflections: age (in years), sex (men; women), marital status [married, living together with spouse; other (divorced; widowed; single; married, living together with spouse)], education [according to the ISCED-97 (12) classification: low, medium, or high education], income poverty [no; yes; according to the threshold of 60% of median household net equivalence income which corresponds to 968 Euro in this study (13)], and asset poverty [no; yes; if asset was 0 to 500 Euro, asset poverty was assumed (13)].

With regard to health-related factors, the following determinants were included in regression analysis: depressive symptoms, cognitive impairment, and chronic conditions. Depressive symptoms were measured using the “depression in old age scale” (DIA-S) (14, 15). This tool includes four items (no or yes in each case). A sum score was computed which ranged from 0 to 4 (higher values reflect more depressive symptoms). Favorable psychometric characteristics have been documented (14, 15). The DemTect (16, 17) was used to assess cognitive impairment. The score ranges from 0 to 18, with higher scores reflecting *lower* cognitive impairment. Moreover, a count score of chronic conditions was generated (for each condition: 0 = absence; 1 = presence). The 19 chronic conditions were as follows: myocardial infarction, heart failure, hypertension, stroke, mental illness, cancer, diabetes, respiratory or pulmonary disease, back pain, gastric or intestinal disease, kidney disease, liver disease, blood disease, joint or bone disease, bladder disease, sleep disorder, eye disease or visual disorder, ear disease or hearing impairment, and neurological disease.

### 2.4 Statistical analysis

First, sample characteristics for the analytical sample are shown. To this end, we calculated the mean with standard deviation (for continuous variables) or the number with percentage (for categorical variables). Since we were particularly interested in the association between financial poverty (in terms of income poverty and asset poverty) and functional impairment, we calculated corresponding effect sizes (Cohen’s *d*). Cohen’s *d* was therefore used to get a better idea of the magnitude of the effect.

Thereafter, multiple linear regressions were conducted to examine the determinants of functional impairment. To address missing data, a full-information maximum likelihood (FIML) approach was used (18). The statistical significance was defined as *p*-value of ≤0.05. Stata 16.1 was used for statistical analyses (Stata Corp., College Station, TX, USA).

## 3 Results

### 3.1 Sample characteristics and effect sizes

Sample characteristics for the analytical sample are shown in Table 1. Average age was 86.5 years (SD: 4.5 years, ranging from 80 to 102 years). Moreover, 50.2% of the individuals were female. In sum, 14.7% of the individuals were classified as income poor and 21.4% of the individuals were classified as asset poor. The average functional impairment score (ADL) was 0.4 (SD: 0.5) and the average instrumental functional impairment score (IADL) was 0.6 (SD: 0.6). Further details are shown in Table 1.

**TABLE 1:** Sample characteristics (*n* = 1,863 individuals).

Variables	Mean (SD)/*N* (%)
**Sex**	
Men	927 (49.8%)
Women	936 (50.2%)
Age	86.5 (4.5)
**Marital status**	
Married, living separated from spouse; widowed; divorced; single	1,107 (59.5%)
Married, living together with spouse	755 (40.5%)
**Educational level (ISCED-97)**	
Low	421 (24.3%)
Medium	929 (53.7%)
High	379 (21.9%)
**Income poverty**	
No	1,187 (85.3%)
Yes	205 (14.7%)
**Asset poverty**	
No	866 (78.6%)
Yes	236 (21.4%)
Depressive symptoms (DIA-S4, ranging from 0 to 4, with higher values corresponding to more depressive symptoms)	0.9 (1.1)
Cognitive impairment (ranging from 0 to 18; with higher values corresponding to lower cognitive impairment)	14.3 (3.9)
Count score: chronic conditions (ranging from 0 to 19, with higher values corresponding to more chronic conditions)	3.5 (2.3)
Functional impairment (ADL; ranging from 0 to 2, with higher values corresponding to higher functional impairment)	0.4 (0.5)
Functional impairment (IADL; ranging from 0 to 2, with higher values corresponding to higher functional impairment)	0.6 (0.7)

It may be worth noting that Cohen’s *d* (in absolute terms) for the association between income poverty and functional impairment was as follows: *d* = 0.34 (with ADL) and *d* = 0.43 (with IADL). Moreover, the association between asset poverty and functional impairment was as follows: *d* = 0.40 (with ADL) and *d* = 0.36 (with IADL).

### 3.2 Regression analysis

Results of multiple linear regressions are shown in Table 2. In the second column, functional impairment (in terms of ADL) was used as dependent variable. In the third column, functional impairment (in terms of IADL) was used as dependent variable. In Table 2, unstandardized beta-coefficients (with robust standard errors in parentheses) are displayed.

**TABLE 2:** Determinants of functional impairment.

Independent variables	Dependent variable: Functional impairment (ADL)	Dependent variable: Functional impairment (IADL)
Sex: women (Ref.: men)	0.06**	0.09**
	(0.02)	(0.03)
Age	0.02***	0.04***
	(0.00)	(0.00)
Marital status: married, living together with spouse (Ref.: married, living separated from spouse; widowed; divorced; single)	0.02	–0.04
	(0.02)	(0.03)
Educational level (ISCED-97): Medium (Ref.: low)	−0.06^+^	–0.06
	(0.03)	(0.04)
High	–0.06	−0.10*
	(0.04)	(0.05)
Income poverty: yes (Ref.: no)	0.09*	0.16**
	(0.05)	(0.05)
Asset poverty: yes (Ref.: no)	0.03	–0.02
	(0.05)	(0.05)
Depressive symptoms (ranging from 0 to 4, with higher values corresponding to more depressive symptoms)	0.12***	0.14***
	(0.01)	(0.01)
Cognitive impairment (ranging from 0 to 18; with higher values corresponding to lower cognitive impairment)	−0.03***	−0.06***
	(0.00)	(0.00)
Count score: chronic conditions (ranging from 0 to 19, with higher values corresponding to more chronic conditions)	0.03***	0.05***
	(0.00)	(0.01)
Constant	−1.51***	−2.36***
	(0.28)	(0.31)
*R* ^2^	0.32	0.44
Observations	1,863	1,863

Results of multiple linear regressions. In the second column, functional impairment (in terms of ADL) was used as dependent variable. In the third column, functional impairment (in terms of IADL) was used as dependent variable. Unstandardized beta-coefficients are displayed; robust standard errors in parentheses; FIML was used to address missing data.

***p < 0.001, **p < 0.01, *p < 0.05, ^+^*p* < 0.10.

*R*^2^ was 0.32 with functional impairment (in terms of ADL) as outcome measure and 0.44 with instrumental functional impairment (in terms of IADL) as outcome measure. The highest variance inflation factor (VIF) was 2.29 (mean VIF: 1.37) suggesting the absence of multicollinearity.

Regressions showed that higher functional impairment was associated with being female (ADL, β = 0.06, *p* < 0.01; IADL, β = 0.09, *p* < 0.01), higher age (ADL, β = 0.02, *p* < 0.001; IADL, β = 0.04, *p* < 0.001), low education (compared to high education: IADL, β = −0.10, *p* < 0.05), the presence of income poverty (ADL, β = 0.09, *p* < 0.05; IADL, β = 0.16, *p* < 0.01), more depressive symptoms (ADL, β = 0.12, *p* < 0.001; IADL, β = 0.14, *p* < 0.001), higher cognitive impairment (ADL, β = −0.03, *p* < 0.001; IADL, β = −0.06, *p* < 0.001), and a higher number of chronic conditions (ADL, β = 0.03, *p* < 0.001; IADL, β = 0.05, *p* < 0.001). In contrast, higher functional impairment (both; ADL and IADL) was neither significantly associated with marital status nor with asset poverty.

## 4 Discussion

Drawing on data from a large, representative sample, our aim was to investigate the determinants of functional impairment among the oldest old. Regressions showed that higher functional impairment was significantly associated with being female, higher age, low education, the presence of income poverty, more depressive symptoms, higher cognitive impairment and a higher number of chronic conditions.

The most striking and innovative result of our study is that the presence of income poverty is associated with a higher risk of functional impairment. Potential mechanisms may be that income poverty is associated with lower internal locus of control (i.e., individuals with a low internal locus of control do not strongly believe in the fact that life’s outcomes are based on their own efforts) (19), low health literacy (20), poor nutrition (21), or other adverse lifestyle-related factors (such as smoking, high alcohol intake, or sedentary behavior) (22, 23) – and could therefore contribute to functional impairment (24). In contrast to that, asset poverty was not significantly associated with functional impairment in our study. At first glance, this may seem counterintuitive. However, asset could be built up through external factors such as inheritances (rather than career success that could ensure a good pension in high age). Thus, asset poverty may not necessarily reflect adverse lifestyle factors that could explain functional impairment.

Beyond that, the association between the sociodemographic factors (i.e., higher age and being female) and functional impairment are well in line with the current literature (5). Furthermore, the association between the health-related factors (depressive symptoms, cognitive impairment, and chronic conditions) and functional impairment is in accordance with existing studies (5–7) – and may be explained by factors such as physical inactivity or reduced social contacts (25, 26).

When assessing our data, it is important to keep in mind several strengths and limitations. It should be emphasized that data from a little-studied population (individuals aged 80 years and over) were used. Furthermore, data were gathered from a sizable, representative sample that also included individuals living in institutions, as opposed to previous research that mostly focused on those who lived in the community. Additionally, both, the outcomes and the determinants were quantified using valid tools. Despite the relatively low response rate, the NRW80+ is regarded as representative of individuals in their 80s who reside in North Rhine-Westphalia (9). The directionality of the associations should be taken with caution due to the cross-sectional design.

## 5 Conclusion

In conclusion, our study identified some determinants of functional impairment among the oldest old. This knowledge may assist in characterizing individuals aged 80 years and over at high risk for functional impairment. Upcoming research particularly based on longitudinal data would be desirable. Moreover, upcoming studies from other areas of the world would be beneficial. Additionally, upcoming research should clarify the underlying mechanisms (e.g., in the association between income poverty and functional impairment). Furthermore, future studies could examine the determinants of functional impairment among the oldest old – stratified by living arrangement (i.e., individuals residing in private households and individuals residing in institutionalized settings).

## Data availability statement

The NRW 80+ data are available *via* gesis. For interested researchers, see: https://search.gesis.org/research_data/ZA7558.

## Ethics statement

The studies involving human participants were reviewed and approved by the Ethics Committee of the Medical Faculty of the University of Cologne (No. 17-169). The patients/participants provided their written informed consent to participate in this study.

## Author contributions

AH: conceptualization, data curation, methodology, project administration, visualization, roles/writing—original draft, writing—review and editing, and formal analysis. H-HK: conceptualization, resources, writing—review and editing, supervision, and visualization. Both authors contributed to the article and approved the submitted version.
